# Distribution Changes in Lichen: A Staple Fallback Food for Yunnan Snub-Nosed Monkey and Their Implications for the Species

**DOI:** 10.3390/biology14101369

**Published:** 2025-10-07

**Authors:** Yuan Zhang, Hanyu Zhu, Lianghua Huang, Xinming He, Sang Ge, Jiandong Lai, Duji Zhaba, Dayong Li, Wancai Xia

**Affiliations:** 1 Key Laboratory of Southwest China Wildlife Resources Conservation (Ministry of Education), China West Normal University, Nanchong 637009, China; zzzzzzzzzy@163.com (Y.Z.); 18084802573@163.com (H.Z.); 980119lsc@163.com (D.L.); 2 Key Laboratory of Conservation Biology of *Rhinopithecus roxellana*, China West Normal University, Nanchong 637009, China; 3 Yunnan Baimaxueshan National Nature Reserve, Diqing 674499, China; 4 Wildlife Forensic Science Service, Kunming 650201, China

**Keywords:** Yunnan snub-nosed monkey, lichen, climate change, BIOMOD2, change in suitable distribution area

## Abstract

**Simple Summary:**

Lichens, as a staple fallback food, play a crucial role in the survival of Yunnan snub-nosed monkey (*Rhinopithecus bieti*). We used the species distribution models (SDMs) to simulate and predict the current and projected (2050) suitable distribution areas of lichens and explored how lichens affect the distribution of Yunnan snub-nosed monkey through spatial pattern analysis. The results showed a high degree of spatial consistency between the suitable distribution areas of Yunnan snub-nosed monkey and lichens, indicating that Yunnan snub-nosed monkey preferred to select the lichens with a large continuous area as their habitat. While the suitable distribution area of lichens in the southern part of the Hengduan Mountain Range is projected to expand by 2050, lichens within the suitable habitat of the Yunnan snub-nosed monkey are at risk of decline in the future. This study provides important insights into the habitat selection of Yunnan snub-nosed monkey and offers a scientific basis and theoretical support for their conservation.

**Abstract:**

Under the background of global climate change, lichens as a staple fallback food source for the endangered Yunnan snub-nosed monkey (*Rhinopithecus bieti*) exert a critical influence on the survival of Yunnan snub-nosed monkey populations through their distribution dynamics. This study focused on the contiguous habitats of the Yunnan snub-nosed monkey in the southern Hengduan Mountains. By species distribution models (SDMs) and landscape pattern analysis, we investigated the changes in suitable habitats of lichens under four Representative Concentration Pathway (RCP) scenarios and their implications for the habitat utilization of the Yunnan snub-nosed monkey until 2050. The results indicate that the current suitable habitat for lichen spans approximately 16,821.96 km^2^, with highly suitable habitats predominantly located in Deqin County and Weixi County. Altitude and vegetation type emerged as primary factors influencing lichen distribution. The overlap rate of suitable habitats between lichens and the Yunnan snub-nosed monkey is 72.24%. Furthermore, the Yunnan snub-nosed monkey exhibits a preference for selecting habitats characterized by the largest patch index (LPI) of lichen distribution. By 2050, the suitable habitat for lichen is projected to marginally increase in the southern Hengduan Mountains, particularly under the RCP 6.0 scenario, by 22.20% compared to the current expansion. However, both the suitable habitat and the LPI of lichen face potential decline within the habitat of the Yunnan snub-nosed monkey. Therefore, we recommend conducting a quantitative investigation into the correlation between the actual productivity of lichen radiata and the population dynamics of Yunnan snub-nosed monkey as a priority. This research will offer a more precise scientific foundation for conservation decision-making for Yunnan snub-nosed monkey.

## 1. Introduction

Against the backdrop of the “Anthropocene”, climate change and the expansion of human activities have exerted a profound impact on global ecosystems, triggering unprecedented ecological crises [1]. The global surface temperature has risen by approximately 1.1 °C compared to the pre-industrial period [2]. This change poses multi-dimensional threats to the survival of wild animals by exacerbating the fragmentation of their habitats and the pressure of resource competition [2,3]. Meanwhile, the process of urbanization and the intensification of land use have led to significant degradation of 75% of the world’s terrestrial ecosystems [4]. This systemic loss of habitats directly threatens 27% of mammals, significantly increasing their risk of extinction [5,6]. These synergistic pressures have catalyzed dynamic adjustments in biogeographical patterns, forcing species to weigh between the degradation of habitat quality and adaptive strategies. Current hypotheses regarding distribution changes emphasize three main driving factors: (1) The anthropogenic disturbance hypothesis: direct human interventions (such as logging and infrastructure expansion) have significantly reduced global biodiversity and the distribution ranges of species [7,8,9]; (2) The landscape alteration hypothesis: changes in landscape and habitat quality caused by the destruction and degradation of natural ecosystems profoundly affect species distribution [10]; (3) The climate change hypothesis: the suitable habitat for species will dynamically adjust with climate change, prompting species to respond to habitat changes caused by climate change [11]. For example, the combined effects of global warming and anthropogenic disturbances have driven species to shift to higher latitudes and altitudes [12]. These hypotheses reveal the profound impacts of climate change and human activities on biodiversity and provide an important theoretical framework for understanding the reshaping of species adaptive landscapes.

Species Distribution Models (SDMs) have emerged as a valuable tool over the past three decades for predicting potential suitable habitat of species [13,14], finding applications in diverse fields such as geography, ecology, and conservation biology [15]. Particularly in the realm of conservation biology, SDMs have played a crucial role in investigating the impacts of climate change on biodiversity. Numerous studies have leveraged SDMs to assess the correlation between habitat suitability and climate change, as well as to forecast changes in habitat suitability under evolving climatic conditions [13,16,17,18]. These models treat species’ habitat adaptation as a constant factor while considering habitat alterations driven by climate change as a variable. By statistically analyzing species site data alongside environmental predictors, SDMs delineate basic and realized niches [19], identify optimal habitats for populations, and offer valuable insights into population responses to climate change [20,21]. Furthermore, SDMs find widespread utility in evaluating conservation efficacy, analyzing biogeographical patterns [22], and facilitating the reintroduction of rare and endangered species [23]. BIOMOD2 is a comprehensive R-based modeling platform that integrates ten algorithms, including regression, classification, and complex models such as GLM, GAM, GBM, CTA, ANN, FDA, SRE, MARS, RF, and MAXENT [13,24]. The ensemble modeling approach in BIOMOD2 improves prediction robustness compared to individual models by leveraging two mechanisms: (1) algorithmic consensus, which involves a weighted average of predictions from models with different structural assumptions [25]; (2) uncertainty quantification, which calculates coefficients of variation among model outputs to identify regions with high prediction inconsistency [26]. Comparative studies have shown that the ensemble model combination in BIOMOD2, as opposed to a single model, can simultaneously incorporate outputs from multiple algorithms, offering more accurate and reliable predictions [27,28].

In primate ecology, fallback food refers to an alternative food source that a species turns to when its primary food supply is limited. The utilization of fallback food is intricately linked to habitat quality and seasonal variations [29,30]. Based on functional distinctions, fallback foods can be categorized into two types: filler fallback foods and staple fallback foods. Filler fallback foods are temporarily utilized during challenging conditions primarily to supplement nutrition or alleviate hunger [31]. For instance, *Pongo pygmaeus* resort to tree cambium as a short-term food source during food scarcity, although it does not constitute their entire diet [32]. On the other hand, staple fallback foods, while nutritionally or palatably inferior to preferred foods, are widely available and require minimal effort to acquire. These foods serve as vital resources for animals during food shortages and can sustain them over extended periods. *Gorilla gorilla* exemplify this by their enduring reliance on fibrous plants for sustenance [33]. The Yunnan snub-nosed monkey (*Rhinopithecus bieti*) is an endangered primate native to China, with a feeding strategy centered on staple fallback food, displaying a significant reliance on lichen [34,35], *Bryoria* spp. and *Usnea longissima*, two lichen species, form the primary dietary components of this species, accounting for approximately 67% of its annual food intake. During the dry season, the consumption of these lichens escalates to over 80% of its total diet [34]. Lichens are typically found hanging from branches in alpine coniferous forests, and are abundant in carbohydrates, cellulose, and hemicellulose [36], providing crucial nutritional benefits to the Yunnan snub-nosed monkey. Despite the challenging digestibility of these compounds for most animals, the monkey effectively utilizes this polysaccharide-rich food source by mechanically breaking down lichen fibers with specialized molars and converting them into digestible volatile fatty acids through a distinctive microbial fermentation process within its S-shaped, compartmentalized stomach [37,38]. Lichens exhibit remarkable ecological adaptations, such as cold and desiccation tolerance, as well as the synthesis of secondary metabolites, allowing them to thrive in high-altitude environments where vascular plant growth is limited [39]. Particularly in winter or dry seasons, lichens stand out as one of the primary accessible resources in high-altitude habitats [40]. Lichens exhibit remarkable ecological adaptations such as cold and desiccation tolerance, as well as the synthesis of secondary metabolites, allowing them to thrive in high-altitude environments with limited vascular plant productivity. It serves as a crucial resource in such habitats, serving as a primary nutrient source to sustain metabolic activity, particularly during harsh winter and dry seasons. This dual function of lichen as both a readily available resource and a vital nutritional source establishes it as a unique staple food reserve in primate feeding research, supporting year-round sustenance.

In the context of global climate change, the sustainability of ecologically specialized animals hinges on the adaptive capacity of their critical resources to climate variations. However, prevailing conservation paradigms and research agendas predominantly emphasize species’ macrohabitat suitability, often neglecting the dynamics of essential resources [41]. Primates, in particular, are heavily reliant on the availability of these crucial resources [30], and alterations in habitat or depletion of key food sources can result in localized extinctions [19,42]. The scarcity of lichen resources, a primary food source for the Yunnan snub-nosed monkey, may compel these primates to expand their foraging areas to meet their nutritional needs, thereby heightening risks of heightened energy expenditure, intraspecies competition, and human–wildlife conflicts [43]. Consequently, the decline in lichen habitat signifies not only a vegetative shift but also an ecological trigger precipitating a survival crisis for the Yunnan snub-nosed monkey population. The conservation status of the Yunnan snub-nosed monkey is intricately linked to the safeguarding of this critical resource. This neglect of dynamic changes in crucial resources poses a significant threat to high-altitude species such as the Yunnan snub-nosed monkey; changes in their crucial resource base can directly impact the distribution patterns of these monkeys in the face of global warming trends [40,42,43]. Notably, lichens have been found to be fragile and vulnerable to human-induced environmental changes, such as the dramatic decline of forests in Europe, which is generally attributed to air pollution, especially sulfur dioxide [44,45]. China is the world’s largest emitter of sulfur dioxide [46], and the Hengduan Mountains are even considered as one of the important carbon sinks in southern and southwestern East Asia [40]. In the context of increasing urbanization and climate change, it is worthwhile to study the distributional changes in staple reserve foods for Yunnan snub-nosed monkey in the Hengduan Mountains.

This study uses the BIOMOD2 (version3.5.1) package developed based on R software (version 4.2.3) to predict the suitable habitat of lichen, the staple fallback food of Yunnan snub-nosed monkey, under the current and four different future climate scenarios, and analyzes the main driving factors affecting changes in lichen’s suitable habitat. In addition, through landscape pattern analysis, it explores the impact of the spatial distribution of lichen within the suitable habitat of Yunnan snub-nosed monkey on their habitat selection. The research results aim to provide scientific guidance for formulating conservation strategies, population recovery, and reintroduction of Yunnan snub-nosed monkey based on the dynamics of staple food resources in the future, offering a theoretical basis and practical support.

## 2. Materials and Methods

### 2.1. Research Area

**Figure 1:**
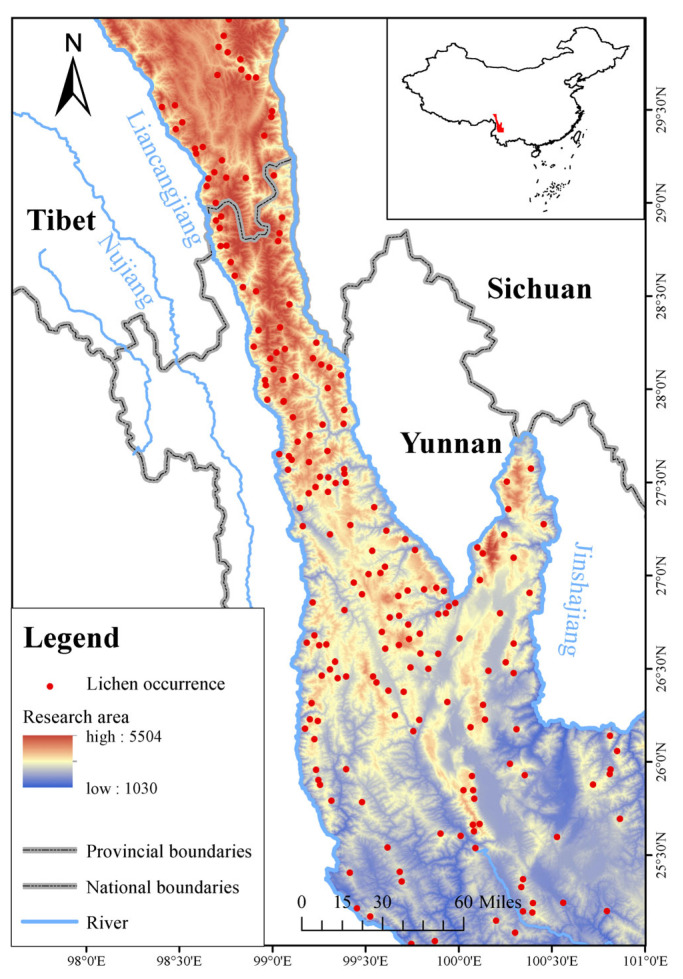
Occurrence records and overview of the research area.

The research area is located in the southern segment of the Hengduan Mountains, a narrow region flanked by the Jinsha River and Lancang River, spanning from 25°30′ N to 101°98′ E, with elevations ranging from 1030 m to 5504 m, encompassing approximately 59,871 km^2^ (Figure 1). The climatic conditions in this locality predominantly consist of a subtropical monsoon climate in northwest Yunnan and a plateau and alpine climate in Tibet, giving rise to intricate and diverse climate gradients. Vegetation types exhibit marked variations along the altitudinal gradient, transitioning from evergreen broad-leaved forests at lower elevations to coniferous forests, deciduous broad-leaved forests, and alpine dark coniferous forests at higher elevations. The research area encompasses all documented habitats of the Yunnan snub-nosed monkey population and represents the primary distribution region of both the Yunnan snub-nosed monkey and their principal food source [47].

### 2.2. Occurrence Collection

We conducted field surveys from June 2022 to March 2025 to collect distribution data on lichen. In the study area, 2396 5 × 5 km grids were generated using ArcGIS, from which 599 grids were randomly selected as survey units. Within each grid, a 2 km sample line was randomly established for on-site investigation. Data on latitude, longitude, altitude, slope, and slope position of lichen along the sample line were recorded. We marked confirmed absence points in habitats such as flowing stone beaches and urban residential areas where lichen’s parasitic host trees were absent. To reduce model errors caused by sampling bias, all marked points were thinned using ArcGIS (version 10.8.2) with a thinning radius of 5 km to ensure that there was only one point in each grid. After screening, we retained 203 occurrence point data of lichen and 389 absence point data. The presence point data was labeled as 1 and the absence point data was labeled as 0 through the Presences.Absences parameter in the R language and converted to csv format for subsequent use in constructing the integrated model.

### 2.3. Environmental Parameters

Based on the physiological and ecological characteristics of lichen, three groups of environmental variables, including climate factors, topographic factors, and vegetation type factors, totaling 23 variables, were selected as the initial environmental predictors for training the SDM (Table 1). Among them, the climate factors include 19 bioclimatic variables (bio1-bio19). These are four integrated concentration and emission scenarios based on Climate Model Intercomparison Project 5 (CMIP5) projections that were used as input parameters of the prediction model for climate change in the 21st century under the influence of human activities. They represent a low-emission scenario (2.6 W·m^−2^, RCP2.6), an intermediate-stabilization scenario (4.5 W·m^−2^, RCP4.5), a medium-emission scenario (6.0 W·m^−2^, RCP6.0), and a high-emission scenario (8.5 W·m^−2^, RCP8.5). The data were sourced from the WorldClim dataset (https://worldclim.org/, accessed on 26 February 2024) with a spatial resolution of 30 arc-seconds. Topographic factors (elevation, slope, and aspect) were extracted from the GDEMV2 30 M resolution digital elevation data obtained from the Geospatial Data Cloud (http://www.gscloud.cn/, accessed on 26 February 2024). Vegetation type data were derived from the 250 m vegetation coverage dataset in the China region released by the National Tibetan Plateau Data Center (http://www.tpdc.ac.cn, accessed on 15 March 2024). The current suitable habitat data of the Yunnan snub-nosed monkey were obtained from previous studies [48]. All data types are raster data. Using the projection transformation tool in ArcGIS 10.8, the projection coordinate systems of all raster data were unified to (WGS_1984_World_Mercator). We extracted the study area using a mask and resampled the spatial resolution to 200 × 200 m, then saved it as ASCII format.

**Figure A1:**
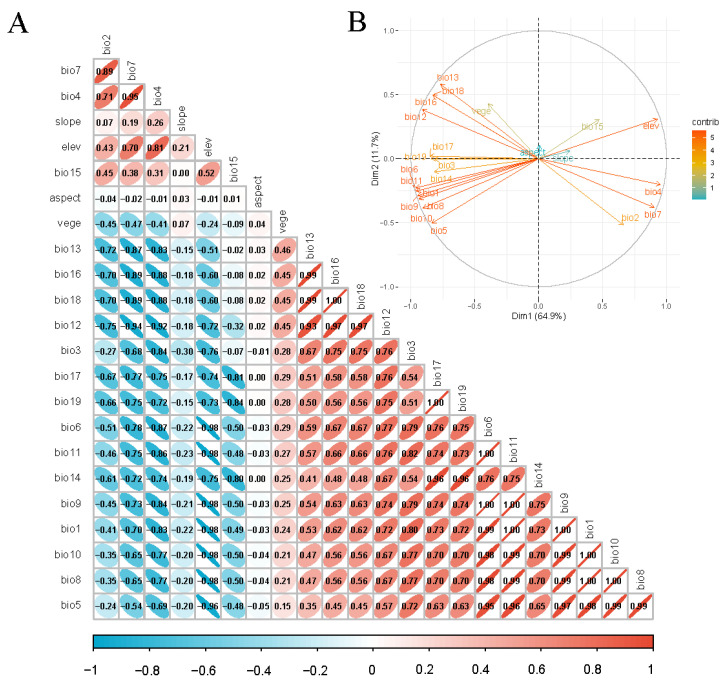
(**A**) Heatmap of Spearman’s rank correlation coefficient between environmental variables; (**B**) Classical Principal Component Analysis (PCA) results for 23 environmental variables.

The 19 bioclimatic variables, such as bio1-bio19, are derived from precipitation and temperature calculations, and there may be correlations and multicollinearity among them, which can lead to deviations in model operation results and a relatively higher AUC value than the actual one [49]. Before constructing the model, we calculated and analyzed the Spearman correlation coefficients among all variables and conducted a classical Principal Component Analysis (PCA) [50]. For a pair of variables with a correlation coefficient |r| ≥ 0.8, the variable with a higher contribution rate to lichen distribution was retained for model construction based on the PCA results (Figure A1). Finally, we selected 8 variables with low correlation and relatively high contribution rates (bio2, bio3, bio12, bio15, Ele, Slo, Asp, and Vege) for further research.

### 2.4. Model Settings and Assessment

When using Biomod2 to analyze the potential habitats of species, the core role of constructing pseudo-absences is to help the model distinguish the environmental differences between the occurrence and non-occurrence areas of species by simulating the environmental background conditions where species truly do not exist [13]. Although we obtained some absence data through field surveys earlier, due to the large sample size of presence data (presence: absence ≈ 1:1.9), the model is at risk of overfitting, which may lead to an overestimation of the range of suitable areas. To balance model performance and computational efficiency, we used the BIOMOD_FormatingData function in the Biomod2 package to import the distribution points of lichen into the model and used PA.nb to control the generation of pseudo-absences (PA). We uniformly generated “pseudo-absence” data three times with 700 points each time, forming a complete species “presence-absence” database (presence: absence ≈ 1:5). This approach avoids excessive simplification or inflation of model prediction results caused by data defects. The generation of pseudo-absences adopted a completely random sampling strategy to improve the computational efficiency of the model.

To improve the prediction accuracy of the model, before combined modeling, we first evaluated the ten algorithms of the BIOMOD2 package using True Skill Statistics (TSS) and Area Under the Receiver Operating Characteristic Curve (AUC). An AUC < 0.6 indicates poor performance, 0.6 ≤ AUC < 0.7 indicates relatively poor performance, 0.7 ≤ AUC < 0.8 indicates average performance, 0.8 ≤ AUC < 0.9 indicates good performance, and 0.9 ≤ AUC < 1 indicates excellent performance. The TSS score ranges from −1 to 1; a value closer to 1 indicates higher prediction accuracy of the model, and a value closer to −1 indicates that the model is more inclined to be random [51]. Different proportions of training set/test set (80%/20%, 75%/25%, 70%/30%) were used to verify the model. The proportion of training set/test set with the highest AUC and TSS values was selected to run the model, and 10 repeated iterations were performed to reduce the uncertainty of the modeling results. We retained models with TSS > 0.7 and AUC > 0.9 and used the BIOMOD_Ensemblemodeling function with the EM.algo parameter to select from four methods for integrated modeling. The options include EMmean (based on the mean probability of the selected models), EMcv (based on the coefficient of variation in probabilities of the selected models, i.e., SD/mean), EMca (based on binary voting of the selected models), and EMwmean (based on evaluation scores), where the probabilities of the selected models are weighted by the evaluation scores obtained during model construction [52]. After running the ensemble model, the obtained results were imported into ArcGIS, the grid values were converted to 0–1000, and the natural breaks method was used to reclassify the layers, dividing them into four suitability levels: unsuitable, lowly suitable, moderately suitable, and highly suitable [53]. The same classification criteria were used to reclassify the prediction results of different future climate scenarios, and the raster calculator built into ArcGIS was used to calculate the area of suitable habitat at each level.

### 2.5. Spatial Correlation Analysis of the Distribution of Yunnan Snub-Nosed Monkey and Lichen

The prediction result files of the current suitable habitat of Yunnan snub-nosed monkey and lichens were imported into ArcGIS to extract the overlapping areas of the two suitable habitats, and the area proportion of lichen suitable habitat in the suitable habitat of Yunnan snub-nosed monkey was calculated by Zonal Statistics.

According to the barrier of road and river and the distribution of Yunnan snub-nosed monkey, the suitable habitat of Yunnan snub-nosed monkey was divided into different areas, and the landscape pattern index of lichen suitable habitat in each area was calculated by FRAGSTATS 4.2 [54]. Four indices were used to evaluate the spatial fragmentation of lichen distribution: patch density (PD), reflecting the number of suitable patches per unit area, indicating strong continuity of lichen distribution; a high PD value indicated severe fragmentation, which directly affected the foraging efficiency of Yunnan snub-nosed monkey [55]; aggregation index (AI), quantifying the spatial concentration degree of lichen patches. The higher the AI value, the higher the concentration degree of food resources, the more obvious the core area that monkeys can use, and the contrary indicates that resources are scattered. Largest patch index (LPI), which characterizes the area proportion of dominant lichen patches, ranges from 0 (no suitable patch) to 100% (all suitable patches); CONTAG describes the spatial connectivity of lichen distribution. The calculation formulas for these indicators are as follows:

Import the results into SPSS (version 27.0.1). Due to the small sample size (n = 15) and the fact that some indices do not conform to the normal distribution as tested by the Shapiro–Wilk test, the Mann–Whitney U test was used to analyze the differences in landscape indices of the suitable habitat of lichens between the patches with and without the distribution of Yunnan snub-nosed monkey. Each region was divided into two groups according to the actual distribution records of monkey groups (present = 1, absent = 0), with the statistical significance level set at *p* ≤ 0.05.

## 3. Results

### 3.1. Model Results and Performance

By comparing the performance of the model for three given training/test set partitioning ratios (80%/20%, 75%/25%, 70%/30%), we found that the 75%/25% partitioning significantly optimized the predictive power of the ensemble model (AUC = 0.993, TSS = 0.912), followed by 10 iterations at that ratio to ensure the stability of the results. Among the ten models based on the BIOMOD2 platform, GLM, GBM, RF, MARS, and MAXENT performed best in predicting the potential suitable habitat of lichen (Table 2). The five high-performance single models were selected, and integrated models were constructed by four integration methods: EMmean, EMcv, EMca, and EMwmean. The results showed that the prediction accuracy of all integrated models was significantly higher than that of single models, and the integrated model constructed by the EMca method performed the best (AUC = 0.912 ± 0.03, TSS = 0.993 ± 0.01), indicating that the ensemble model can predict the potential suitable habitat of lichen more accurately.

### 3.2. Importance of Environmental Variables

Elevation was the most important environmental factor (Ele, 0.664), and its influence was significantly higher than other variables. Vegetation type was the second most important factor, and its contribution rate was 0.338. Among the climatic variables, the annual mean precipitation (bio12, 0.118) and the ratio of temperature difference between day and night to annual temperature difference (bio3, 0.113) had moderate contributions, while the monthly mean temperature difference between day and night (bio2, 0.037) and the variance of precipitation (bio15, 0.081) had relatively limited contributions, and the slope (Slo, 0.039) and aspect (Asp, 0.041) had no obvious effects on the distribution pattern of lichen (Table 3).

### 3.3. Potential Suitable Habitat of Lichen in Present and Future

#### 3.3.1. Potential Suitable Habitat of Lichen

The prediction results show that the potential suitable habitat of lichen is about 16821.96 km^2^, accounting for 28.10% of the total area of the study area, of which the low-suitable habitat is about 5911.49 km^2^, the medium suitable habitat is about 3692.74 km ^2^, and the high-suitable habitat is about 7217.73 km^2^. The high-suitable habitat of lichen is mainly concentrated in the Deqin County and Weixi County in the middle of the study area, accounting for 12.06% of the total area of the study area. The middle-suitable area (6.17%) was distributed in the periphery of the high-suitable area in a ring shape and overlapped with the transition zone of alpine dark coniferous forest and broad-leaved mixed forest. The low-suitable area (9.87%) is mainly distributed in the secondary forest area with an altitude of 2200–2600 m, the unsuitable areas (71.9%) were mainly located in areas with high human activities such as Xiangyun County, Midu County, Binchuan County, Dali City, and Weishan Yi and Hui Autonomous County County, which were below 2200 m in elevation, and in areas without arboreal hosts, such as snow-covered mountains and alpine rhyolite beaches, which were in the southern part of Yanjing County and the central part of Mangkang County, which were above 4000 m in elevation.

#### 3.3.2. Potential Suitable Habitat of Lichen in the Future

**Figure 2:**
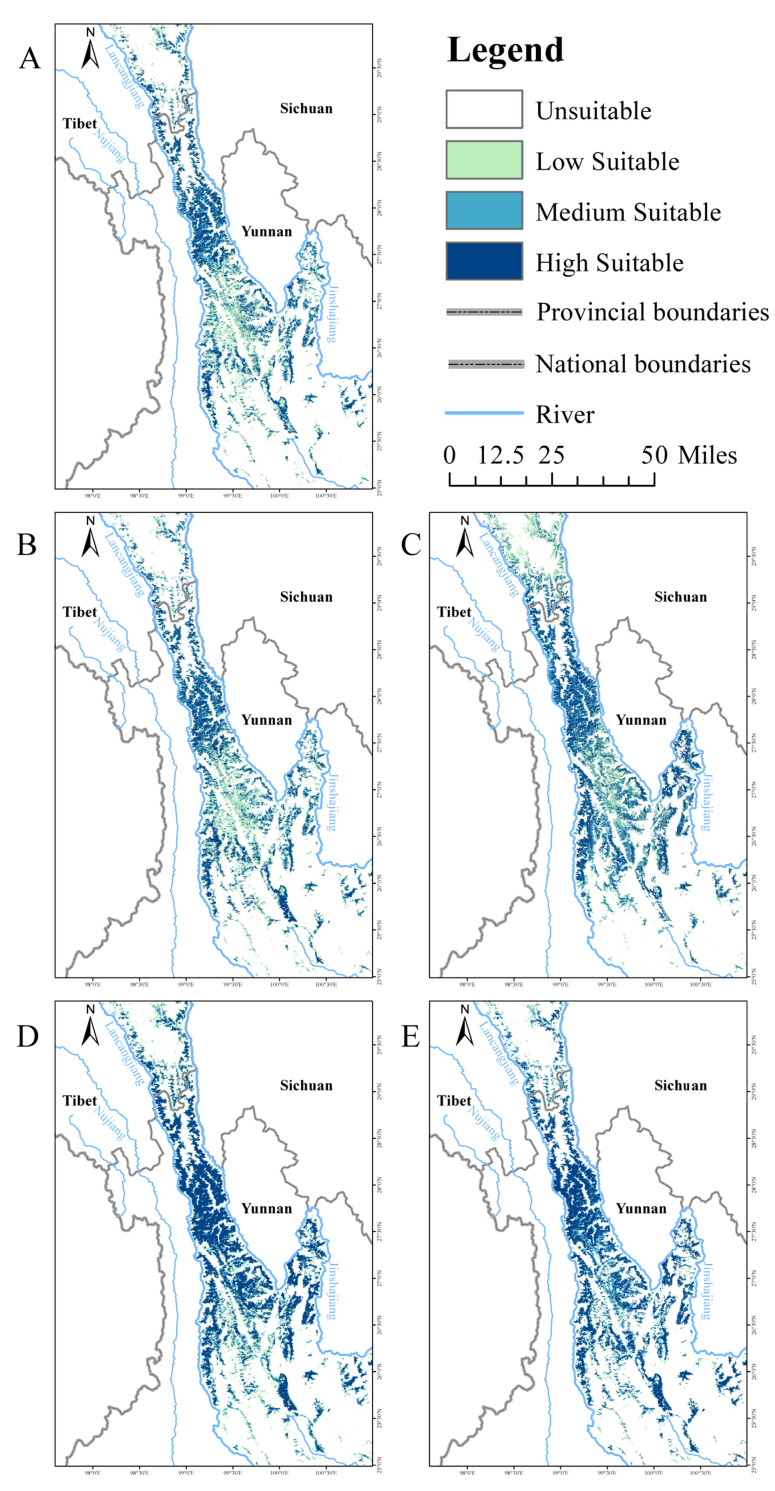
Distribution of suitable habitat for lichen under different climate scenarios in the future. (**A**) Current, (**B**) RCP2.6, (**C**) RCP4.5, (**D**) RCP6.0, (**E**) RCP8.5.

Compared to the current situation, the suitable distribution pattern of lichen remains largely unchanged across future climate scenarios, with its suitable habitat exhibiting varying degrees of expansion. The average expansion proportions of the suitable habitat are 1.30% under RCP2.6, 18.81% under RCP4.5, 22.20% under RCP6.0, and 1.62% under RCP8.5. In terms of high-suitability habitats, the RCP6.0 scenario demonstrates the most significant expansion over time, followed by RCP8.5 and RCP4.5, while RCP2.6 remains stable, with high-suitability habitats still concentrated in the Deqin County and Weixi County. Notably, under the RCP8.5 scenario, although the suitable habitat of lichen shows only a slight expansion, habitat suitability increases markedly, with low-suitability habitats transitioning to medium- and high-suitability habitats. Specifically, low-suitability habitats decrease by 65.91% compared to the current situation, medium-suitability habitats increase by 17.68%, and high-suitability habitats increase by 48.71% (Figure 2).

### 3.4. Correlation Between the Distribution of Lichen and Yunnan Snub-Nosed Monkey

**Figure 3:**
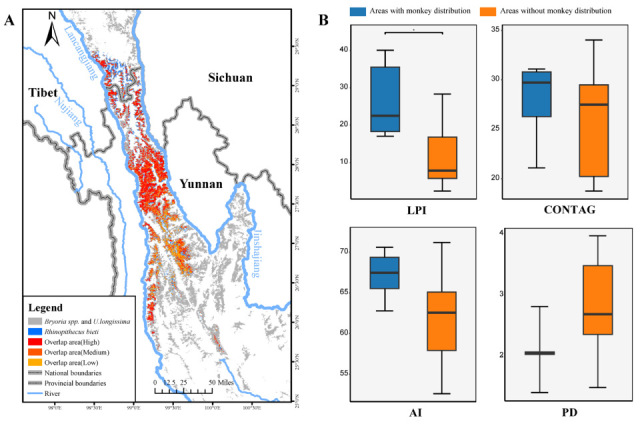
(**A**) Overlapping areas of suitable ranges of Yunnan snub-nosed monkey and lichen. (**B**) Comparison of landscape pattern metrics of lichen in areas with and without monkey distribution. "*" indicates a significant difference between the two groups, i.e., "*p* < 0.05".

At present, the suitable habitat of the Yunnan snub-nosed monkey is about 7412.82 km^2^, in which the area overlapping with the suitable habitat of lichen reaches 5354.75 km^2^, accounting for 72.24% of the total suitable habitat of the Yunnan snub-nosed monkey, in which the suitable habitat overlapping with lichen is 3275.95 km^2^, accounting for 61.23% of the total overlapping area; the suitable habitat overlapping with lichen is 2078.81 km^2^, accounting for 38.77% of the total overlapping area (Figure 3A).

Among the 15 suitable habitats of the Yunnan snub-nosed monkey, six areas are inhabited by one or more groups of this species. Analysis of landscape pattern indices revealed that CONTAG (*p* = 0.456), PD (*p* = 0.088), and AI (*p* = 0.066) did not exert a significant influence on the distribution of Yunnan snub-nosed monkey. However, the largest patch index (LPI) of lichen radiata had a significant impact on the distribution of Yunnan snub-nosed monkey (*p* < 0.05). Specifically, the LPI of plaques associated with Yunnan snub-nosed monkey was significantly higher (Mean ± SD = 26.48 ± 9.65) compared to plaques without this species’ distribution (Mean ± SD = 13.83 ± 12.66) (Figure 3B).

### 3.5. Effect of Lichen Distribution on Yunnan Snub-Nosed Monkey in the Future

**Figure 4:**
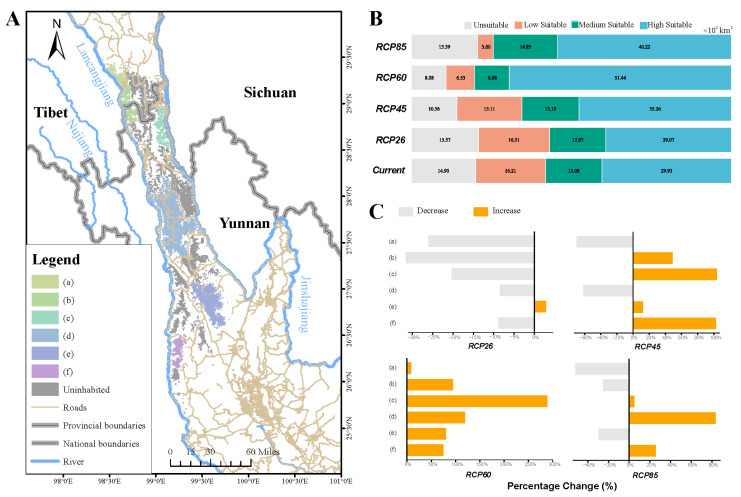
(**A**) The current suitable habitat area for the Yunnan snub-nosed monkey. (**B**) The area of lichen in suitable habitat of the Yunnan snub-nosed monkey under the current and future climate scenarios. (**C**) Changes in LPI in areas with monkey distribution. (a) Zhina, Xiaochangdu monkey groups; (b) Milaka, Bamei monkey groups; (c) Wuyapuya monkey group; (d) Cikakong, Guilong, Shiba, Yongan, Anyi, Xiangguqing, Gehuaqing, Shikuadi monkey groups; (e) Jinsichang monkey group; (f) Changyanshan, Lashashan, Longmashan monkey groups.

We further explored the overlap rate of suitable habitat of the Yunnan snub-nosed monkey (Figure 4A) and suitable habitat of lichen under different climatic scenarios in the future and the change in LPI of lichen landscape pattern in the monkey area. Compared with the present situation, the overlap ratio between Yunnan snub-nosed monkey and lichen in the suitable habitat of RCP4.5 (+5.86%) and RCP6.0 (+9.20%) showed an expansion trend, and a slight contraction occurred in RCP2.6 (−0.90%) and RCP8.5 (−0.65%). Under the RCP8.5 scenario, the loss areas were all low-suitable habitat, and the middle- and high-suitable habitat expanded by 28.20% compared with the current situation, the overall suitability of the habitat was improved (Figure 4B).

From the change in landscape pattern in the suitable habitat of lichen, the LPI of monkey suitable habitat increased under RCP6.0 (Mean ± SD = 52.06 ± 19.69) and RCP8.5 (Mean ± SD = 27.44 ± 20.69) scenarios and decreased under RCP2.6 (Mean ± SD = 22.20 ± 8.23) and RCP4.5 (Mean ± SD = 26.08 ± 9.75) scenarios. Under the RCP2.6 scenario, only the LPI of the Jinsichang monkey group increased, and the rest of the areas decreased; under the RCP4.5 scenario, the LPI of Zhina, Xiaochangdu, Cikatun, Guilong, Shiba, Yongan, Anyi, Xiangguqing, Gehuaqing, and Shikuadi decreased, and the rest of the areas increased; under the RCP6.0 scenario, the LPI of all monkey group suitable habitat increased; and under the RCP8.5 scenario, the LPI of Zhina, Xiaochangdu, Milaka, Bamei, and Jinsichang decreased, and the rest of the areas increased (Figure 4C). 

## 4. Discussion

This study utilized the BIOMOD2 ensemble modeling to accurately simulate the current and future distribution of suitable habitat for lichen, the staple fallback food for the Yunnan snub-nosed monkey. Our models demonstrated high predictive accuracy (AUC = 0.993, TSS = 0.912) and revealed a high degree of spatial overlap between the suitable habitats of the lichen and the monkeys, confirming that the LPI of the lichen is the primary factor influencing habitat selection by the Yunnan snub-nosed monkey. The results indicate that although the suitable habitat for lichen is projected to expand under various RCP scenarios, critical risks—including habitat fragmentation and severe degradation of landscape patterns—threaten certain monkey populations. As expected, this research provides a scientific foundation and practical support for informing future conservation strategies, population recovery, and reintroduction programs for the Yunnan snub-nosed monkey based on the dynamics of their staple fallback food resources.

### 4.1. Model Prediction Accuracy

Based on the BIOMOD2 integrated modeling platform, this study integrated climate, terrain, and vegetation types, and other environmental variables to accurately simulate the current and future suitable area distribution of lichen. The platform builds an ensemble model [56] by integrating multiple algorithmic models with the same initial dataset and *p*-parameterization, significantly reducing prediction bias [12] caused by differences in individual models, and using a mutual correction mechanism between models to improve result reliability [57]. Model validation shows (Table 2) that AUC and TSS values of the integrated model constructed by screening and integration are better than those of all single models (0.993, 0.912), and the comprehensive performance is good, which can predict the suitable habitat of lichen more accurately. Furthermore, in traditional species distribution models, pseudo-nonexistent points are usually generated randomly within the study area by algorithms. However, due to the parasitic habits of lichens, even if the climatic conditions are suitable, areas lacking trees, such as high mountain flow stone beaches, bare rocks or artificial construction areas cannot support its survival [36,58]. Therefore, combining the physiological constraints of lichens, this study combined the “non-tree cover area” as a real non-existent point into the pseudo-nonexistent point dataset and the algorithm generation points, effectively suppressed the false high prediction of the model for the climate suitable but habitat mismatch area, and improved the ecological rationality of the simulation results and the problem of excessive niche expansion caused by ignoring the physiological constraints of species in traditional SDMs. BIOMOD2 has been successfully applied to plant prediction, such as peony [59], fritillaria [60], and larch [61]. In addition, the model prediction is based on the assumption that the environment is stable, so the prediction results can only represent the current situation in a certain period of time. If the environment changes greatly in a short period of time, the applicability of the results will decrease, which is also the limitation of the model. Therefore, this study mainly discusses the distribution pattern of lichen, a staple food of Yunnan snub-nosed monkey, on a large scale.

### 4.2. Temporal and Spatial Variation and Driving Factors of Suitable Habitat of Lichen

Rapid climate change has led to the redistribution of many species [52]. Lichens as a staple fallback food for Yunnan snub-nosed monkey are highly dependent on microclimate conditions of high humidity, low pollution, and stable hydrothermal conditions [62]. In this study, the BIOMOD2 ensemble model was used to simulate and predict the current and future potential suitable habitat of lichen. The current suitable habitat of lichen was highly overlapped with the existing habitat of Yunnan snub-nosed monkey (Figure 3A), mainly distributed in the virgin forest in the Deqin County and Weixi County. The spatial distribution pattern of lichen under four different RCP scenarios in the future has no obvious change compared with the current situation, and the suitable habitat has an expanding trend along the current suitable habitat to varying degrees (Figure 2). Elevation (Ele) is the dominant factor affecting the distribution of lichen. With the increase in altitude, atmospheric pressure and air density decrease. Low atmospheric pressure and air density inhibit the retention of pollutants. Meanwhile, low temperature and low air density in high altitude areas lead to faster wind speed under the same pressure gradient, which helps to disperse pollutants and form a clean air environment [63]. Moreover, the internal water content of the lichens is in equilibrium with the surrounding atmosphere, so their activity is very dynamic, often varying on time scales ranging from minutes to hours [64]. The cascade effect of altitude, isotherm (bio2), and annual mean precipitation (bio13) results in the cloud belt with stable hydrothermal conditions providing ideal habitat for it and the correct combination of microclimate factors [65], periodic triggering of lichen vapor-induced reactivation [58], continuously maintaining its normal physiological metabolic state. On the contrary, due to high temperature, low humidity, and intense human activities, the air pollution in the dry and hot valley area at low altitude is serious, and there is almost no lichen distribution, which also confirms its sensitivity to the ecological environment [64]. Vegetation type (Vege) is the basic limiting factor affecting the distribution of lichen due to its parasitic habit, and its distribution is greatly limited by the growth range of host trees. However, under the background of global warming, the greenhouse effect drives a large number of vegetation in high altitude areas to expand [57,66], which may promote the synchronous expansion of suitable lichen areas. In addition, the symbiotic algae of lichen are extremely sensitive to air humidity and temperature [67]. The greenhouse effect leads to the shortening of the snow cover cycle at high altitude, which may prolong the annual photosynthetic cycle of lichen [68], while the increase in atmospheric CO^2^ concentration will increase the carbon fixation efficiency of its symbiotic algae [67], which will promote the accumulation of lichen biomass and further expansion of suitable areas.

The vegetation type data included in the simulation prediction of lichen suitable area under different climate scenarios in the future are the current annual normalized vegetation index data. From now to 2050, the vegetation type in the study area will remain unchanged, and only climate change will be considered. Lichens may benefit from climate change.

### 4.3. Food Security Potential and Sustainable Utilization Challenge of Yunnan Snub-Nosed Monkey

Lichens as a staple fallback food for Yunnan snub-nosed monkey are considered to be a specific habitat dependent evolution [40]. This study confirms the high spatial consistency of the Yunnan snub-nosed monkey and lichen, and its spatial distribution pattern has a profound impact on habitat use of Yunnan snub-nosed monkey, and more than 72% of the suitable areas overlap (Figure 3), confirming the high spatial consistency of the suitable habitat of lichen and Yunnan snub-nosed monkey [43], providing food security for the population. In winter, when food is scarce and cold, Yunnan snub-nosed monkey will increase foraging input to compensate for the decrease in food quantity and quality and energy pressure caused by cold temperature or reduce the activity time of the monkey group to reduce energy loss [69]. Therefore, in the alpine woodland with a cold climate and food shortage, Yunnan snub-nosed monkey will tend to choose the more developed area of continuous patch of LPI as habitat [23], while a large area of continuous forage can significantly reduce foraging energy consumption. Reducing the ecological cost of group maintenance, thereby allowing the formation of “supergroups,” is also consistent with standard primate foraging theory [70].

In the future, 2050, the suitable habitat of lichen will expand under different climatic conditions, but the lichen resources in the existing habitat of the Yunnan snub-nosed monkey will show differential response. Under RCP2.6 and RCP8.5 scenarios, the suitable habitat of lichen in monkey populations experienced a slight shrinkage, while in the RCP8.5 scenario, the shrinkage area was mainly low-suitable area, and the proportion of medium and high-suitable areas increased significantly. The enhancement of habitat suitability may offset some negative effects caused by the suitable habitat from the increase in lichen biomass. It should be noted that landscape pattern analysis showed that 6 of the 15 habitat areas with monkey populations significantly increased their LPI under the RCP6.0 scenario, while different monkey populations faced LPI attenuation risks under other scenarios. The LPI of Zhina and Xiaochangdu populations at high altitude decreased in all scenarios except RCP6.0. The Xiaochangdu and Zhina regions are inhabited by the highest altitude Yunnan snub-nosed monkey population, and their habitats are restricted by plateau mountain climate. The vegetation is mainly dark coniferous forest, which leads to the diversity of food resources being significantly lower than that of low altitude regions. The proportion of lichen in the annual diet of monkeys in this region is as high as 80% [40], which constitutes absolute nutrient dependence, the cascading effect triggered by the range contraction of the lichen habitat and the decrease of the LPI will most likely lead to the risk of regional collapse of the Yunnan snub-nosed monkey population in the region.

During our research, we found that the area and LPI of the overlapping suitable habitat in which the Yunnan snub-nosed monkey and lichens in the Nalizhuse mountain area are next to those of the Xiangguqing and Gehuaqing core areas, and most of this region is located within the Baima Snow Mountain National Nature Reserve. However, historical monitoring and current investigation have not recorded the activity trace of Yunnan snub-nosed monkey in this area. The area is closed off by multiple geographical barriers: the east is cut off by Jinsha River Canyon, the north is adjacent to Xilan Line and Baima Snow Mountain Line Highway, the northwest is a high mountain with perennial snow, and the southwest and south are surrounded by transportation infrastructure such as the thick cartoon highway and Qixia Highway. This kind of natural and artificial compound barrier completely blocks the migration corridor of primates, forming ecological islands [71]. Based on its abundant food resources and suitable climatic conditions, the main color mountain range in Nali can be used as a priority candidate for the introduction of Yunnan snub-nosed monkey and the establishment of a new population of Yunnan snub-nosed monkey.

From the results, climate change impacts for lichens and Yunnan snub-nosed monkey are not necessarily entirely negative. Therefore, how to seize the opportunities brought by climate change and reduce the risks brought by climate change deserves our careful consideration. As far as Yunnan snub-nosed monkey are concerned, the suitable habitat of lichen will expand under different climatic conditions in the future, which theoretically provides favorable conditions for its population maintenance. However, we must carefully assess this potential for practical feasibility. As the main food source of Yunnan snub-nosed monkey, the physiological and ecological characteristics of lichens may seriously restrict their practical availability [67]. Grueter et al. (2009) found through long-term observations that the regeneration rate of lichen is extremely slow, and it usually takes decades to fully recover from feeding pressure on lichen [43]. At present, the population of Yunnan snub-nosed monkey is highly fragmented, and some areas of its habitat have obvious range contraction and edge limitation due to human activities such as tourism development. This habitat fragmentation makes the already slow growing lichen resources face greater foraging pressure. With the continuous increase in monkey population in the area, if its activity range cannot be effectively expanded, the feeding pressure will inevitably exceed the annual growth of lichen and eventually lead to irreversible degradation of local ecological resources. Crucially, the expansion of the lichen habitat does not mean that Yunnan snub-nosed monkey can actually use these resources. Habitat fragmentation is increasing with population growth and land use change. Previous studies have also shown that the actual distribution range of the Yunnan snub-nosed monkey has shrunk sharply under the condition of suitable habitat expansion. Many areas meeting the survival conditions of the Yunnan snub-nosed monkey, such as food resources, cannot be used by the Yunnan snub-nosed monkey due to roads, buildings, and rivers. This “habitat trap” phenomenon may weaken the potential benefits brought by climate change.

## 5. Conclusions

Under the combined effects of climate change and greenhouse gas emissions, the suitable habitat of lichen, the main food source of the Yunnan snub-nosed monkey, showed a continuous expansion trend. Based on landscape pattern analysis, we found that the LPI of lichen spatial distribution was a key driver for habitat selection of high-quality food for Yunnan snub-nosed monkey. Although climate change may bring more potential food resources to Yunnan snub-nosed monkey, the slow regeneration of lichen, the threat of air pollution, and habitat accessibility barriers pose a triple challenge. In the future, monitoring of air pollution and restoration of habitat connectivity should be strengthened, and the relationship between actual productivity of lichen radiata and population dynamics of Yunnan snub-nosed monkey should be quantified to provide a more accurate scientific basis for conservation decision-making.

## Figures and Tables

**Table 1:** Interpretation and filtering of 23 environment variables.

Variable Type	Unit	Description	Selection
Climatic variables	bio1	Annual mean temperature (°C)	
	bio2	Mean diurnal temperature range (°C)	√
	bio3	Isothermality (bio2/bio7) (×100)	√
	bio4	Temperature seasonality (standard deviation × 100)	
	bio5	Maximum temperature of warmest month (°C)	
	bio6	Minimum temperature of coldest month (°C)	
	bio7	Temperature annual range (°C)	
	bio8	Mean temperature of wettest quarter (°C)	
	bio9	Mean temperature of driest quarter (°C)	
	bio10	Mean temperature of warmest quarter (°C)	
	bio11	Mean temperature of coldest quarter (°C)	
	bio12	Annual precipitation (mm)	√
	bio13	Precipitation of wettest month (mm)	
	bio14	Precipitation of driest month (mm)	
	bio15	Precipitation seasonality (mm)	√
	bio16	Precipitation of wettest quarter (mm)	
	bio17	Precipitation of driest quarter (mm)	
	bio18	Precipitation of warmest quarter (mm)	
	bio19	Precipitation of coldest quarter (mm)	
Topographical variables	Asp	Aspect	√
	Ele	Elevation	√
	Slo	Slope	√
Vegetation variables	Vege	Vegetation type	√

**Table 2:** TSS and AUC values for 10 models and model selection.

Model Variable		TSS	AUC	Model Selection
Single model	GLM	0.766 ± 0.02	0.919 ± 0.01	√
	GBM	0.806 ± 0.03	0.932 ± 0.02	√
	GAM	N/A	N/A	
	CTA	0.746 ± 0.03	0.877 ± 0.02	
	ANN	0.621 ± 0.01	0.835 ± 0.03	
	SRE	0.488 ± 0.05	0.744 ± 0.04	
	FDA	0.730 ± 0.03	0.901 ± 0.01	
	MARS	0.750 ± 0.03	0.912 ± 0.02	√
	RF	0.809 ± 0.03	0.938 ± 0.01	√
	MAXENT	0.728 ± 0.05	0.918 ± 0.03	√
Integrated model	EMmean	0.867 ± 0.03	0.969 ± 0.03	
	EMca	0.912 ± 0.03	0.993 ± 0.01	√
	Emcv	N/A	N/A	
	EMwmean	0.869 ± 0.03	0.971 ± 0.02	

Note: N/A indicates that the model evaluation metric is not available.

**Table 3:** Contribution of distributional variables of lichen in the study area.

Variable	Aspect	bio2	bio3	bio12	bio15	Ele	Slo	Vege
**Contributions**	0.039	0.037	0.113	0.118	0.081	0.664	0.041	0.338

## Data Availability

The data presented in this study are not public but are available on request from the corresponding author due to them being part of an ongoing research project.
